# Comprehensive genomic assessment of available probiotics in India: A framework for safety and functionality evaluation

**DOI:** 10.6026/973206300211163

**Published:** 2025-05-31

**Authors:** Swati Shrivastava, Nidhi Sharma, Abhishek Sharma, Shubhi Gupta

**Affiliations:** 1 Department of Biochemistry, Government Medical College, Datia, Madhya Pradesh, India; 2 Department of Microbiology, Government Medical College, Datia, Madhya Pradesh, India

**Keywords:** Probiotics, whole genome sequencing, safety assessment, antimicrobial resistance, genomic analysis, functional properties

## Abstract

Probiotic strains, defined as live microorganisms that confer health benefits to the host when administered in adequate amounts, are widely incorporated into commercial products. Therefore, assessing the safety and functional attributes of 20 commercially available probiotic strains using complete genome sequencing is of immediate importance. Our analysis revealed that 15% of the products were mislabeled, containing species different from those declared on the label. Further, 12% of the tested strains harbored transferable antimicrobial resistance genes. However, 8% Bacillus species contained virulence-associated genes. Nonetheless probiotic-related genes linked to acid and bile tolerance were consistently found in Lactobacillus strains. These findings underscore the urgent need for standardized genomic safety assessments prior to the market approval of probiotic products.

## Background:

The definition of probiotics describes these "live microorganisms" which deliver health advantages to the host body when given in appropriate doses. Probiotics currently serve as dietary supplements and functional food ingredients throughout the worldwide market. The probiotic market shows rapid international growth because people worldwide understand better how their digestive system and immune system work. Sales of commercial probiotics continue to rise while complete security evaluations of their product strains persist with inconsistent standards which creates potential safety and performance-related issues [1]. The current approach to evaluate probiotic safety depends mainly on identifying microorganisms taxonomically while defining characteristics through phenotypic examinations using the principle of historical safe usage. This method does not consider how variations at the strain level affect both safeties together with functionality profiles. Modern research shows that taxonomically linked strains of probiotics exhibit notable genomic variations that influence their functional properties while impacting security aspects and tendencies for genetic information exchanges [2, 3- 4]. The technological advancement of Whole genome sequencing (WGS) provides extensive information about probiotic strain genomic structures by performing extensive microbial analysis WGS serves valuable functions in food safety assessments and the European Food Safety Authority (EFSA) recommends its application and use for assessing microorganisms meant for the food production chain The EFSA delivered recommendations through their 2024 statement regarding WGS analysis reporting procedures for regulatory purposes and established its value in taxonomic identification and genetic modification detection and genes of concern identification [5].

Safety assessments for probiotic strains must evaluate their antimicrobial resistance genes together with their mobility potential along with virulence properties, toxin production abilities and elements capable of horizontal gene transfer. The safety evaluation of microbial cultures can be assessed through Pariza *et al.*'s decision tree approach which includes 13 questions that evaluate safety aspects in a systematic manner [1]. Microorganisms with both enzyme production and probiotic properties have potential applications in food and animal feed industries [6]. Genomic analysis helps researchers find genetic features which contribute to probiotic functionality while operating above safety evaluation needs. Research reveals that beneficial genes determine acid and bile tolerance functions together with the ability to adhere to mucosal surfaces and generate useful metabolites as well as demonstrate immunological effects [7]. The identification of functional traits at the genomic level serves as an essential requirement for validating probiotic claims in addition to explaining their operational mechanisms [8]. The application of WGS technology exists in multiple investigations performing probiotic strain characterization. The analysis of *Bacillus subtilis* strain PLSSC through WGS showed its safety properties and probiotic characteristics by demonstrating that it lacks functional AMR genes along with virulence factors [9, 10]. Research on Lactobacillus strains through genomic comparison identified key genes which show relation to probiotic capabilities such as acid and bile tolerance as well as antimicrobial effects and adhesive surface proteins [3]. The application of WGS-based technologies has been minimally studied for investigating commercial probiotic products. Various antimicrobial resistance elements and toxic genetic components detected in some commercial *Bacillus* probiotics warrant complete genetic testing as a requirement prior to market authorization [8]. Various studies discover that commercial probiotics sold with inaccurate taxonomic labelling do not contain their advertised strains which reveal deficient quality control systems in commercial production [4]. Therefore, it is of interest to develop frameworks for the safety and operational competence of commercially available probiotic strains using complete genome sequencing procedures.

## Materials and Methods:

## Study design and sample collection:

Twenty commercial probiotic products were randomly selected from retail outlets in major metropolitan areas between January and March 2024. Selection criteria included representation of diverse bacterial genera commonly used as probiotics (*Lactobacillus*, *Bifidobacterium*, Bacillus and *Saccharomyces*) and various product formulations (capsules, tablets, powders and fermented foods). Products were transported to the laboratory under appropriate temperature conditions and stored according to manufacturer recommendations until processing.

## Strain isolation and identification:

Microbial strains were isolated from commercial products using selective media appropriate for each genus. For capsules and tablets, the contents were aseptically removed and serially diluted in sterile phosphate-buffered saline. Appropriate dilutions were plated on Man Rogosa Sharpe (MRS) agar for Lactobacillus species, *Bifidobacterium* selective medium (BSM) for *Bifidobacterium* species and nutrient agar for Bacillus species. Plates were incubated under appropriate conditions (aerobic, anaerobic, or microaerophilic) at optimal temperatures for each genus. Representative colonies were selected and purified through three successive streaking on the respective media.

## DNA extraction and whole genome sequencing:

Genomic DNA was extracted from pure cultures using the DNeasy Blood & Tissue Kit (Qiagen, Germany) following the manufacturer's protocol with modifications for Gram-positive bacteria [12]. DNA quality and quantity were assessed using a NanoDrop spectrophotometer and Qubit fluorometer. For each strain, both short-read and long-read sequencing approaches were employed. Illumina sequencing libraries were prepared using the Nextera XT DNA Library Preparation Kit and sequenced on an Illumina NovaSeq 6000 platform with 2x150 bp paired-end reads. Oxford Nanopore sequencing was performed using the Ligation Sequencing Kit and MinION device following the manufacturer's instructions.

## Genome assembly and annotation:

Raw reads from both sequencing platforms were quality-filtered using FastQC and Trimmomatic. A hybrid assembly approach combining Illumina short reads and Nanopore long reads was implemented using Unicycler v0.4.8. The completeness of assembled genomes was assessed using BUSCO (Benchmarking Universal Single-Copy Orthologs). Genome annotation was performed using Prokka v1.14.6, supplemented with specialized databases for probiotic-associated genes. Taxonomic verification was conducted using 16S rRNA gene analysis, average nucleotide identity (ANI) and core genome phylogeny.

## Safety assessment analysis:

The safety assessment of assembled genomes consisted of analyzing genetic elements for safety using the framework provided by Pariza *et al.* [1]. The ResFinder program along with CARD detected genes which conferring antimicrobial resistance. The detection of virulence factors utilized VirulenceFinder along with VFDB and customized databases from the researched literature. Multiple mobile genetic elements discovered the presence of plasmids transposons and insertion sequences using IslandViewer PHASTER and ICEberg. An assessment of identified AMR gene transferability capability relied on analyzing their genomic environment together with their linkages to mobile elements.

## Functional properties analysis:

Genes linked to probiotic activity identification occurred by conducting literature studies and database research. Studies revealed two important categories of genes which include acid and bile tolerance pathways in addition to mucosal adherence systems and antimicrobial defence operations as well as immunity-regulating mechanisms [12]. The assembled genomes underwent both pathway analysis and custom BLAST searches to determine and define functional elements.

## Statistical analysis:

Genomic characteristics were compared across genera and products using descriptive statistics. Pearson's correlation coefficient was calculated to assess relationships between genomic features and labelled probiotic claims. Hierarchical clustering based on gene presence/absence patterns was performed to identify similarities among analyzed strains. All statistical analyses were conducted using R v4.1.0, with a significance threshold of p<0.05.

## Results:

The whole genome sequencing of 20 commercial probiotic products yielded high-quality assemblies for 24 distinct strains (some products contained multiple strains). Genome completeness, assessed by BUSCO, ranged from 97.2% to 99.8%, indicating the high quality of the assembled genomes. The basic genomic characteristics of the sequenced strains are presented in Table 1. Taxonomic discrepancies between label claims and WGS-based identification were observed in 3 out of 24 strains (12.5%). Most notably, strain P07-L, labelled as Lactobacillus acidophilus, was identified as *Limosilactobacillus fermentum* based on ANI and core genome phylogeny. Similarly, strain P10-S, marketed as *Saccharomyces boulardii* and was genetically indistinguishable from *Saccharomyces cerevisiae*, consistent with current taxonomic understanding that *S. boulardii* is a strain of *S. cerevisiae* rather than a distinct species. The safety assessment of the probiotic strains based on WGS analysis revealed several significant findings (Table 2). Antimicrobial resistance (AMR) genes were detected in 9 out of 24 strains (37.5%), with tetracycline resistance genes (tetM, tetW, tetS) being the most prevalent. However, only 3 strains (12.5%) harboured AMR genes associated with mobile genetic elements, indicating potential for horizontal gene transfer. These included strain P11-B (*Bacillus subtilis*) with an ermB gene on a plasmid, strain P18-B (Bacillus clausii) with a tetL gene flanked by transposon-related sequences, and strain P23-B (*Bacillus licheniformis*) with a catB gene on a putative genomic island.

Virulence factors of concern were identified in 2 Bacillus strains (8.3% of total). Strain P18-B (*B. clausii*) contained two haemolytic factors with significant homology to known hemolysins, while strain P23-B (*B. licheniformis*) harboured cerA and cerB genes associated with cereolysin production. However, functional analysis suggested that these factors might have attenuated activity compared to their counterparts in pathogenic species. No enterotoxin genes (nheA, nheB, nheC, hblC, hblD, hblA) or emetic toxin genes (cesB) were detected in any of the Bacillus strains. Mobile genetic elements were identified in 8 strains (33.3%), predominantly insertion sequences of various families. Three strains contained larger mobile elements: a plasmid in strain P11-B, a transposon in strain P18-B, and a genomic island in strain P23-B. Strain P10-S (*S. cerevisiae*/*S. boulardii*) exhibited Ty2 and Ty5 retrotransposons, consistent with previous reports for this species. The genomic analysis revealed numerous genes associated with probiotic functionality across the analyzed strains. All Lactobacillus strains harbored genes related to acid tolerance (gadB, gadC) and bile resistance (bsh), consistent with their ability to survive gastrointestinal transit. Adhesion-related genes, including those encoding mucus-binding proteins, fibronectin-binding proteins, and S-layer proteins, were identified in 18 strains (75%), with considerable variation in the number and types of adhesion factors across species. Antimicrobial compound production capability, indicated by the presence of bacteriocin gene clusters or enzymatic systems for organic acid and hydrogen peroxide production, was identified in 20 strains (83.3%). Notably, the L. plantarum strains (P04-L and P24-L) contained complete plantaricin biosynthetic gene clusters, while *B. subtilis* (P11-B) harbored genes for subtilin production. Genes involved in the biosynthesis of beneficial metabolites, including folate, riboflavin, and GABA, were detected in various strains, particularly those belonging to Lactobacillus and *Bifidobacterium* genera. Experimental genomic comparisons demonstrated important genetic difference patterns in probiotic factors at the strain level even within species. Genomic evaluation of *L. plantarum* P04-L and P24-L demonstrated variations in the genetic elements affecting adhesion capacity as well as exopolysaccharide biosynthesis cluster activities thus demonstrating why specific strain assessments matter3. Two Bacillus strains were examined and *B. subtilis* (P11-B) acquired the most probiotic genes while *B. coagulans* (P12-B) had fewer.

## Discussion:

This research makes a detailed contribution to the genetic analysis of commercial probiotic bacteria by revealing their biological classification together with security evaluations alongside beneficial characteristics. Whole genome sequencing proves an effective method to assess probiotics but its usefulness depends on standardized evaluation frameworks which guarantee both safety and efficiency standards. The correct identification of commercial probiotic microorganisms showed differences in 12.5% of strains between package information and genuine microbial varieties. Research data supports the existence of mislabelling in commercial probiotic products according to previous findings. The reclassification of P07-L strain from Lactobacillus acidophilus to *Limosilactobacillusfermentum* exposes major product mislabelling which affects both product effectiveness and buyer trust. The probiotic features alongside health advantages of *L. acidophilus* and *L. fermentum* could lead to altered performance effects in the related product. The situation confirms that manufacturers must use proven taxonomic verification systems which surpass classic phenotypic methods2. The genetic identification of *Saccharomyces boulardii* (strain P10-S) as identical to *S. cerevisiae* matches today's taxonomic classifications while factually showing how manufacturers market the organism as a separate species instead of a strain of *S. cerevisiae* [4]. The safety assessment findings revealed several noteworthy patterns regarding antimicrobial resistance genes. While AMR genes were detected in 37.5% of strains, only 12.5% harboured potentially transferable resistance determinants associated with mobile genetic elements. This distinction is crucial, as the European Food Safety Authority (EFSA) and other regulatory bodies primarily focus on transferable resistance as a safety concern for probiotic strains. The presence of ermB on a plasmid in the *B. subtilis* strain (P11-B) is particularly concerning given the clinical importance of macrolide antibiotics. Similarly, the tetL gene associated with a transposon in strain P18-B (*B. clausii*) represents a potential transfer risk, though experimental verification of transfer capability is needed for definitive risk assessment [10, 11]. Regarding virulence factors, the identification of haemolytic factors in *B. clausii* (P18-B) and cereolysin-related genes in *B. licheniformis* (P23-B) warrants careful consideration. While these factors show sequence homology to known virulence determinants, their clinical significance in probiotic strains remains debated [8].

Pediococcus acidilactici SMVDUDB2, isolated from the traditional fermented cheese Kalarei, shows strong probiotic potential due to its high survival in acidic and bile salt conditions, along with its ability to inhibit various pathogenic bacteria [6]. Nevertheless, monitoring of biogenic amine production capability should be incorporated into probiotic safety assessments, particularly for strains intended for fermented food applications. The functional genomic analysis revealed extensive genetic repertoires associated with probiotic properties across the analyzed strains. All Lactobacillus strains harboured acid and bile tolerance genes, consistent with their ecological adaptation to gastrointestinal environments. Whole-genome sequencing helps in evaluating both the safety and functional properties of probiotic bacterial strains. Some probiotic strains can naturally produce beneficial compounds like GABA, which may contribute to health-promoting effects [7]. The antimicrobial compound production potential identified in most strains highlights an important mechanism through which probiotics may confer health benefits. The complete plantaricin gene clusters in *L. plantarum* strains and subtilin genes in *B. subtilis* align with previous genomic characterizations of these species [12, 13- 14]. Similarly, the potential for beneficial metabolite production, particularly GABA, folate, and riboflavin, suggests additional mechanisms by which these strains may promote host health [6]. The observed strain-level variations in probiotic-associated genetic elements, even among strains of the same species, underscore the limitations of species-level safety assessments and support the need for strain-specific genomic evaluation. This finding is consistent with the comparative genomic analysis of Lactobacillus strains by Martinez *et al.* who reported substantial variation in probiotic-related genes across strains of *L. delbrueckii* [3]. Similarly, Diaz *et al.* demonstrated significant genomic diversity among *B. coagulans* strains, affecting their probiotic properties and safety profiles [10]. Our study contains various limitations which need to be acknowledged. Our ability to link genomic data to observable traits was impeded because the researchers did not confirm the functional expression of detected genes experimentally. The analysis only examined the probiotic genera that currently exist in marketplaces despite the fact that these categories may not fully represent the whole scope of potential probiotic candidates. The methodological framework together with analytic procedure proven in this study serves as a useful model for WGS-based assessments of commercial probiotics in future research. This study discloses critical information that affects the regulatory management process of probiotic products. The authors advocate for robust genomic examinations as a mandatory prerequisite for market authorization while indicating that assessment methods might not provide adequate protection for Bacillus and other probiotic genera. The existing decision tree model proposed serves as a strong foundation yet requires addition of precise genomic analysis to inspect the transferable AMR genes and virulence factors and mobile genetic elements. Quality control practices alongside proper labelling during probiotic production gain added significance because of the observed taxonomic inconsistencies [15, 16].

## Conclusion:

The broad genomic examination of commercially available probiotics through whole genome sequencing proves essential for confirming taxonomy along with safety testing while defining product functions. The identification of taxonomic discrepancies in 12.5% of strains and potentially transferable antimicrobial resistance genes in another 12.5% highlights the need for more rigorous quality control and safety evaluation of probiotic products.

## Figures and Tables

**Table 1:** Genomic characteristics of commercial probiotic strains

Strain ID	Genus/Species (Label)	Genus/Species (WGS)	Genome Size (Mb)	GC Content (%)	No. of Contigs	No. of Coding Sequences
P01-L	Lactobacillus acidophilus	Lactobacillus acidophilus	1.98	34.7	2	1,864
P02-L	Lactobacillus casei	Lactobacillus casei	2.92	46.6	3	2,771
P03-B	Bifidobacterium longum	Bifidobacterium longum	2.39	60.3	1	1,992
P04-L	Lactobacillus plantarum	Lactobacillus plantarum	3.24	44.5	4	3,057
P05-B	Bifidobacterium bifidum	Bifidobacterium bifidum	2.21	62.7	2	1,796
P06-L	Lactobacillus rhamnosus	Lactobacillus rhamnosus	3.01	46.8	2	2,834
P07-L	Lactobacillus acidophilus	Limosilactobacillusfermentum*	2.1	51.5	3	1,987
P08-B	Bifidobacteriumanimalis	Bifidobacteriumanimalis	1.94	60.5	1	1,603
P09-L	Lactobacillus helveticus	Lactobacillus helveticus	2.08	37.1	2	2,068
P10-S	*Saccharomyces boulardii*	Saccharomyces cerevisiae**	12.16	38.3	17	5,885
P11-B	*Bacillus subtilis*	*Bacillus subtilis*	4.21	43.5	1	4,232
P12-B	Bacillus coagulans	Bacillus coagulans	3.07	47.3	1	2,985
*Misidentified strain;
taxonomic reclassification suggested
**Confirmed to be *S. cerevisiae* rather
than the distinct species *S. boulardii* as claimed

**Table 2:** Safety assessment of commercial probiotic strains

Strain ID	AMR Genes (Transferable)*	Virulence Factors of Concern**	Mobile Elements	Toxin-Related Genes	Biogenic Amine Synthesis
P01-L	None	None	IS30 family (2)	None	None
P02-L	tetW (No)	None	None	None	None
P03-B	None	None	None	None	None
P04-L	None	None	IS256 family (1)	None	tyrosine decarboxylase
P05-B	None	None	None	None	None
P06-L	None	None	IS3 family (3)	None	None
P07-L	None	None	None	None	None
P08-B	tetW (No)	None	None	None	None
P09-L	None	None	None	None	None
P10-S	None	None	Ty2, Ty5 elements	None	None
P11-B	ermB (Yes)	None	Plasmid (1)	None	None
P12-B	None	None	None	None	None
*Transferability assessment based on genomic
context and association with mobile elements
**Virulence factors considered of
concern based on literature evidence
